# Prions from Sporadic Creutzfeldt-Jakob Disease Patients Propagate as Strain Mixtures

**DOI:** 10.1128/mBio.00393-20

**Published:** 2020-06-16

**Authors:** Hervé Cassard, Alvina Huor, Juan-Carlos Espinosa, Jean-Yves Douet, Severine Lugan, Naima Aron, Didier Vilette, Marie-Bernadette Delisle, Alba Marín-Moreno, Patrice Peran, Vincent Beringue, Juan Maria Torres, James W. Ironside, Olivier Andreoletti

**Affiliations:** a UMR INRA ENVT 1225, Interactions Hôte Agent Pathogène, Ecole Nationale Vétérinaire de Toulouse, Toulouse, France; b Centro de Investigación en Sanidad Animal (INIA-CISA), Valdeolmos, Madrid, Spain; c INSERM U 1214 TONIC, CHU Purpan, Toulouse, France; d Virologie et Immunologie Moléculaires, Domaine de Vilvert, Jouy-en-Josas, France; e National Creutzfeldt-Jakob Disease Research & Surveillance Unit, Centre for Clinical Brain Sciences, University of Edinburgh, Western General Hospital, Edinburgh, United Kingdom; National Institutes of Health

**Keywords:** prion, sporadic Creutzfeldt-Jakob disease, strain diversity, evolution, diversity, prions, sCJD, strains

## Abstract

Sporadic Creutzfeldt-Jakob disease (sCJD) cases are currently classified according to the methionine/valine polymorphism at codon 129 of the *PRNP* gene and the proteinase K-digested abnormal prion protein (PrP^res^) isoform identified by Western blotting (type 1 or type 2). Converging evidence led to the view that MM/MV1, VV/MV2, and VV1 and MM2 sCJD cases are caused by distinct prion strains. However, in a significant proportion of sCJD patients, both type 1 and type 2 PrP^res^ were reported to accumulate in the brain, which raised questions about the diversity of sCJD prion strains and the coexistence of two prion strains in the same patient. In this study, a panel of sCJD brain isolates (*n* = 29) that displayed either a single or mixed type 1/type 2 PrP^res^ were transmitted into human-PrP-expressing mice (tgHu). These bioassays demonstrated that two distinct prion strains (M1^CJD^ and V2^CJD^) were associated with the development of sCJD in MM1/MV1 and VV2/MV2 patients. However, in about 35% of the investigated VV and MV cases, transmission results were consistent with the presence of both M1^CJD^ and V2^CJD^ strains, including in patients who displayed a “pure” type 1 or type 2 PrP^res^. The use of a highly sensitive prion *in vitro* amplification technique that specifically probes the V2^CJD^ strain revealed the presence of the V2^CJD^ prion in more than 80% of the investigated isolates, including isolates that propagated as a pure M1^CJD^ strain in tgHu. These results demonstrate that at least two sCJD prion strains can be present in a single patient.

## Abstract (precis)

sCJD occurrence is currently assumed to result from spontaneous and stochastic formation of a misfolded PrP nucleus in the brains of affected patients. This original nucleus then recruits and converts nascent PrP^C^ into PrP^Sc^, leading to the propagation of prions in the patient’s brain. Our study demonstrates the coexistence of two prion strains in the brains of a majority of the 23 sCJD patients investigated. The relative proportion of these sCJD strains varied both between patients and between brain areas in a single patient. These findings strongly support the view that the replication of an sCJD prion strain in the brain of a patient can result in the propagation of different prion strain subpopulations. Beyond its conceptual importance for our understanding of prion strain properties and evolution, the sCJD strain mixture phenomenon and its frequency among patients have important implications for the development of therapeutic strategies for prion diseases.

## INTRODUCTION

Transmissible spongiform encephalopathies (TSEs), otherwise known as prion diseases, are neurodegenerative disorders affecting a large spectrum of mammalian species that share similar characteristics, including a long incubation period in acquired forms (which in humans may be measured in decades) and a progressive clinical course resulting in death (1). A key event in the pathogenesis of TSEs is the conversion of the normal cellular prion protein (PrP^C^, which is encoded by the *PRNP* gene) into an abnormal disease-associated isoform (PrP^Sc^) in the tissues of infected individuals. PrP^C^ is completely degraded after controlled digestion with proteinase K (PK) in the presence of nondenaturing detergents. In contrast, PrP^Sc^ is N-terminally truncated under such conditions, resulting in a PK-resistant core termed PrP^res^ (2).

The most common form of human TSE is an idiopathic disorder named sporadic Creutzfeldt-Jakob disease (sCJD). In sCJD, two major PrP^res^ isoforms have been described by Western blot (WB) analysis: in type 1 PrP^res^, the apparent molecular weight of the unglycosylated fragment is 21 kDa, while in type 2, it is 19 kDa (3). In sCJD patients, the polymorphism at codon 129 of the prion protein gene (methionine [M]/valine [V]) and the PrP^res^ type (type 1/type 2) are major determinants of the clinical and neuropathological phenotypes of the disease (3, 4).

The currently identified clinical and neuropathological subtypes of sCJD mainly result from codon 129 genotype/PrP^TSE^ type combinations (MM1, VV1, MM2, and VV2), with some exceptions. MM1 and MV1 cases are phenotypically indistinguishable and therefore merged in a single subtype, MM/MV1. VV2 and VV1 constitute two phenotypically distinct subgroups. MV2 cases can be split into three categories according to the predominant presence of cortical lesions (MV2C), presence of kuru type amyloid plaques (MV2K), or a cooccurrence of these features (MV2K+C) (5). Similarly, the MM2 group is divided into two subtypes based on the very distinctive cortical and thalamic histopathology observed in the two groups (MM2-cortical or MM2C and MM2-thalamic or MM2T). Finally, a variant of the MM1 subtype characterized by the presence of widespread PrP amyloid plaques in the white matter has been recognized and designated p-MM1 (6). Based on these observations, it was proposed that the different sCJD phenotype observed in patients (sCJD subtypes) could be associated with the propagation of different sCJD agents (strains) in patients.

In past decades, the poor transmissibility of sCJD brain isolates to conventional mouse models precluded the characterization of sCJD prion biological diversity. This problem was the consequence of the transmission barrier phenomenon that naturally limits the propagation of prions between hosts with different PrP^C^ amino acid sequences (7). Mice genetically engineered to express human PrP (tgHu) in the absence of endogenous mouse PrP^C^ now allow the propagation of human CJD prions without an apparent transmission barrier (8–11).

The main previous study on the characterization of prion strain diversity in sCJD consisted of the transmission of a panel of six sCJD isolates in tgHu mice expressing the Met129 or Val129 human PrP variant at physiological levels (knock-in mice) (12). The bioassay results from the study of Bishop et al. (12) were consistent with the existence of four distinct sCJD prion strains that corresponded to MM/MV type 1, MM type 2 (cortical), MV/VV type 2 and VV type 1 isolates. These data further supported the contention that the combination of the dimorphism at codon 129 of the human *PRNP* gene and the PrP^res^ WB pattern (type 1 or type 2) in the brain tissues of patients provides valuable indications on the nature of prion strains responsible for the disease (12). However, in up to 35% of sCJD patients, both type 1 and type 2 PrP^res^ can be observed in either the same or different brain areas (13, 14). These observations raised questions concerning the actual diversity of sCJD strains and/or the potential coexistence of two or more prion strains in sCJD patients.

In the study presented here, a panel of brain homogenates from 23 sCJD cases originating in three different countries (France, United Kingdom [UK], and Spain) was transmitted to mice that expressed methionine 129 and/or valine 129 human PrP (15). These inocula included samples from individuals that were MM, MV, and VV with respect to the human PrP genotype and displayed either a pure type 1 or type 2 PrP^res^ profile or both type 1 and type 2 PrP^res^ types in their brain. These bioassays demonstrated that two distinct prion strains, named here as M1^CJD^ and V2^CJD^, were preferentially associated with the development of sCJD in MM1/MV1 and VV2/MV2 patients, respectively. However, in more than 50% of the investigated cases, bioassay and/or prion *in vitro* protein misfolding cyclic amplification (PMCA) revealed the presence of both M1^CJD^ and V2^CJD^ prion strains in the sCJD isolates. These results demonstrate that multiple sCJD prion strains can propagate in a single patient. The existence and the frequency of strain mixture phenomenon question the concepts that are currently used to explain the spontaneous occurrence of prions in human sCJD.

## RESULTS

Twenty-three sCJD cases were selected on the basis of their PrP^res^ Western blot (WB) profile and their genotype at codon 129 of the *PRNP* gene. The selected cases originated from three different countries (France, Spain, and UK) and corresponded to MM1, MV1, VV2, and MV2 sCJD cases, which together account for more than 95% of the sCJD cases reported each year by surveillance systems in Europe (5). For each case, the neuropathological data were reviewed to determine the potential particularities in their neuropathological profile. This review indicated that all three identified subtypes of MV2 cases (C, K, and C+K) were part of the panel of isolates examined here (Table 1)

**TABLE 1:** Transmission of sporadic CJD isolates (10% frontal cortex homogenates) into mice expressing the human PrP (methionine, valine, or methionine/valine at codon 129)^*a*^

Isolate				tgMet						tgMet/Val						tgVal					
				Passage 1			Passage 2			Passage 1			Passage 2			Passage 1			Passage 2		
Case	Codon 129^*b*^	Type^*c*^	Orig^*d*^	D/I^*e*^	IncubP^*f*^	PrP^res^ type	D/I	IncubP	PrP^res^ type	D/I	IncubP	PrP^res^ type	D/I	IncubP	PrP^res^ type	D/I	IncubP	PrP^res^ type	D/I	IncubP	PrP^res^ type
1	MM	1	Fr	6/6	210 ± 17	1	6/6	219 ± 8	1	6/6	243 ± 14	1	6/6	241 ± 3	1	6/6	337 ± 14	1	6/6	265 ± 5	1
2			Fr	6/6	221 ± 7	1	6/6	200 ± 11	1	6/6	258 ± 3	1	6/6	242 ± 4	1	6/6	338 ± 28	1	6/6	281 ± 5	1
3			Fr	6/6	250 ± 3	1	6/6	192 ± 8	1	6/6	258 ± 3	1	6/6	244 ± 4	1	6/6	338 ± 28	1	6/6	271 ± 8	1
4			Fr	6/6	230 ± 13	1	6/6	201 ± 11	1	6/6	267 ± 10	1	6/6	232 ± 8	1	6/6	336 ± 8	1	6/6	284 ± 5	1
5			Sp	6/6	211 ± 14	1	6/6	195 ± 13	1	6/6	255 ± 28	1	6/6	245 ± 13	1	6/6	317 ± 10	1	6/6	295 ± 21	1

6	VV	2	Fr	6/6	609 ± 61	1	6/6	480 ± 65	1	6/6	639 ± 28	1	6/6	550 ± 28	1	6/6	191 ± 16	2	6/6	165 ± 4	2
7			Fr	6/6	618 ± 60	1	6/6	509 ± 95	1	6/6	588 ± 74	1	6/6	494 ± 36	1	6/6	168 ± 12	2	6/6	169 ± 12	2
8			Fr	6/6	626 ± 85	1	6/6	548 ± 24	1	6/6	702 ± 19	1	6/6	576 ± 56	1	6/6	198 ± 7	2	6/6	170 ± 7	2
9			Sp	6/6	507 ± 85	1	6/6	428 ± 37	1	6/6	542 ± 63	1		NA		6/6	228 ± 7	2	6/6	228 ± 14	2
10			Sp	6/6	522 ± 36	1	6/6	469 ± 45	1		NA			ND		6/6	194 ± 11	2	6/6	195 ± 14	2
11			Fr	6/6	521 ± 65	1	6/6	216 ± 1	1	6/6	541 ± 109	1	6/6	251 ± 4	1	6/6	188 ± 13	2	6/6	166 ± 6	2

12	MV	1	Fr	6/6	276 ± 19	1	6/6	205 ± 7	1	6/6	321 ± 19	1	6/6	246 ± 7	1	6/6	296 ± 13	1	6/6	282 ± 9	1
13			UK	6/6	197 ± 6	1	6/6	209 ± 17	1	6/6	261 ± 6	1	6/6	247 ± 6	1	6/6	289 ± 13	1	6/6	289 ± 8	1
14			UK	6/6	190 ± 22	1	6/6	208 ± 7	1	6/6	270 ± 14	1	6/6	242 ± 4	1	6/6	284 ± 10	1	6/6	282 ± 6	1
15			Fr	6/6	202 ± 9	1	6/6	206 ± 9	1	6/6	264 ± 8	1	6/6	232 ± 2	1	6/6	258 ± 33	2	6/6	184 ± 6	2
16			Fr	6/6	205 ± 16	1	6/6	220 ± 13	1	6/6	233 ± 4	1	6/6	253 ± 3	1	6/6	232 ± 62	2	6/6	172 ± 12	2

17	MV	2c	UK	6/6	578 ± 28	1	6/6	492 ± 70	1	6/6	631 ± 51	1	6/6	467 ± 28	1	6/6	219 ± 17	2	6/6	174 ± 3	2
18		2k	UK	6/6	533 ± 15	1	6/6	482 ± 14	1	6/6	572 ± 17	1	6/6	485 ± 68	1	6/6	180 ± 9	2	6/6	169 ± 6	2
19		2c+k	UK	6/6	470 ± 23	1	6/6	211 ± 12	1	6/6	628 ± 25	1		NA		6/6	184 ± 9	2	6/6	180 ± 6	2
20		2k	Fr	6/6	375 ± 45	1	6/6	205 ± 5	1	6/6	552 ± 78	1	6/6	248 ± 8	1	6/6	184 ± 9	2	6/6	180 ± 6	2

a Transgenic mice that express the Met_129_ (tgMet) or Val_129_ (tgVal) human PrP and their crossbreed (tgMet/Val) were inoculated intracerebrally (20 μl per mouse) with 20 sporadic Creutzfeldt-Jakob (sCJD) brain tissue homogenates from patients originating from three different countries. The sCJD patients displayed different *PRNP* genotypes at codon 129 and PrP^res^ Western blot isoforms (type 1 or type 2). After the first passage, brain tissue from clinically affected mice were pooled and used for a second passage in the same line. The PrP^res^ WB isoforms (type 1 or type 2) identified in mouse brains are reported for each one of the two passages.

b The sCJD patients displayed different *PRNP* genotypes at codon 129 (MM, homozygous Met_129_; VV, homozygous Val_129_; MV, heterozygous Met/Val_129_).

c 2c, cortical subtype of MV2 sCJD; 2k, kuru plaque subtype of MV2 sCJD; 2c+k, combined cortical and kuru plaque subtype of MV2 sCJD.

d Orig, originating country: France (Fr), Spain (Sp), or the United Kingdom (UK).

e D/I, number of diseased mice/number of inoculated mice.

f IncubP, incubation period (time to death in days). The incubation periods are shown as means ± standard deviations. NA, not available; ND, not done.

Among the selected cases, the first subset (cases 1 to 20) displayed a single PrP^res^ Western blot signature (type 1 or type 2) in the five different brain areas tested. A single brain area (frontal cortex) from each of these cases was used in transmission experiments (Table 1). The second subset (cases 21 to 23) corresponded to sCJD cases that showed mixed type 1/type 2 PrP^res^ signatures in either the same brain area or a different brain area. Three brain areas from each of these cases were selected for bioassay (see Table 3).

The 29 resulting isolates were transmitted (two iterative passages) to mice homozygous for either methionine (tgMet) or valine (tgVal) at codon 129 of human PrP and to their F1 cross (tgMet/Val). All of these different tgHu PrP mouse lines have been shown to express approximately 4-fold-more PrP^C^ in their brain than human brain tissue (15).

### Identification of two dominant distinct sCJD strains.

**FIG 1:**
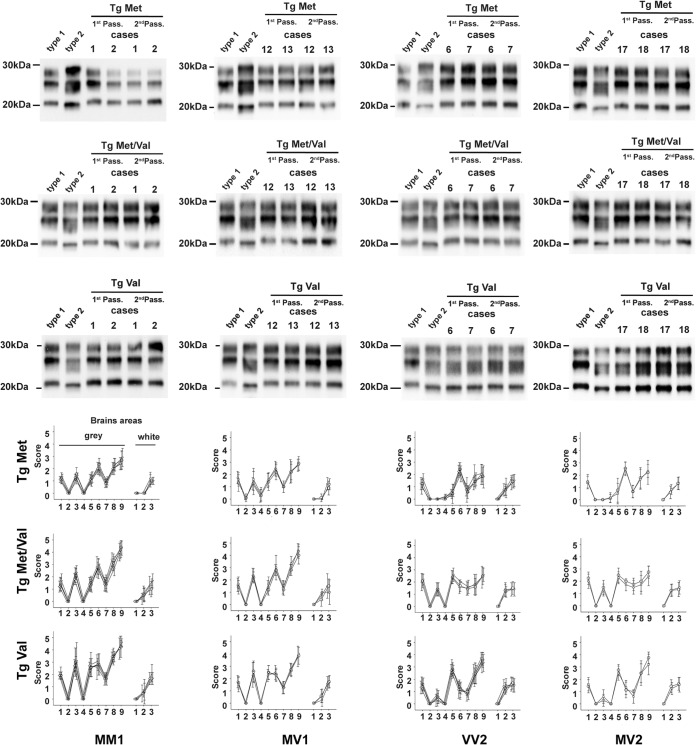
PrP^res^ Western blot profiles (top) and vacuolar lesion profiles (bottom) in the brains of mice expressing human PrP inoculated with sporadic CJD (sCJD). *y* score indicates intensity of vacuolar lesions; *x* axis indicates standard brain gray areas (1 to 9) and white areas (1 to 3). Transgenic mice that express the Met_129_ (tgMet), Val_129_ (tgVal) human PrP and their crossbreed (tgMet/Val) were inoculated intracerebrally (6 mice, 20 μl per mouse) with a 10% brain homogenate (frontal cortex) from sCJD cases that were Met_129_ (MM) homozygotes, Val_129_ (VV) homozygotes, or Met/VAl_129_ (MV) heterozygotes and displayed either a pure type 1 or a pure type 2 PK-resistant PrP (PrP^res^) Western blot (WB) isoform (Table 1). Two iterative passages were performed for each line (Table 1). After the second passage and in each mouse line, the isoform (type 1/type 2) of the PrP^res^ was determined in the mouse brain by sodium dodecyl sulfate-polyacrylamide gel electrophoresis (SDS-PAGE) and WB with the anti-PrP monoclonal antibody Sha31 (epitope YEDRYYRE). A PrP^res^ type 1 isoform (MM1 sCJD isolate) and type 2 isoform (VV2 sCJD isolate) were included as controls on each gel. The WB results obtained in each line are reported in Table 1. In parallel, standardized vacuolar lesion profiles were established in the brains of the same mice. Symbols: in the MM1 lesion profile graphs, ▽, case 1; ▵, case 2; ○, case 3; ◊, case 4; □, case 5; in the MV1 lesion profile graphs, ▵, case 12; ○, case 13; □, case 14; in the VV2 lesion profile graphs, ▽, case 6; ▵, case 7; ○, case 8; ◊, case 9; □, case 10; in the MV2 lesion profile graphs, ○, case 17; □, case 18.

All the MM1sCJD isolates (*n* = 5) and 3 out of 5 MV1 sCJD isolates displayed a short incubation period in tgMet mice (about 200 days postinoculation [dpi]), an intermediate incubation period in tgMet/Val mice (about 250 dpi), and a longer one in tgVal mice (about 300 dpi) (Table 1). Irrespective of the inoculated mouse line, a type 1 PrP^res^ was observed in the brains of the inoculated animals (Table 1 and Fig. 1). We named this transmission pattern M1^CJD^.

The transmission of 5 out of 6 investigated VV2 isolates and 2 out of 4 MV2 isolates was associated with a short incubation period in tgVal mice (about 180 dpi) and a long incubation period in tgMet and tgMet/Val mice (480 to 600 dpi). The inoculated tgMet and tgMet/Val mice accumulated a type 1 PrP^res^ in their brain, while type 2 PrP^res^ was observed in tgVal brains (Fig. 1). This transmission pattern clearly differed from M1^CJD^ and was named V2^CJD^.

In affected tgMet, tgMet/Val, and tgVal mice, a vacuolar lesion profile (see Fig. 3) was established by histological examination of the brain. In each of the three mouse lines, the isolates that displayed an M1^CJD^ transmission pattern showed similar lesion profiles (Fig. 1). Similarly, all isolates displaying a V2 transmission pattern showed the same lesion profile in the tgMet, tgMet/Val, and tgVal mouse lines (Fig. 1). However, the lesion profile associated with the M1 isolates clearly differed from the profile associated with the V2 isolates. Collectively, these results strongly supported the contention that two different prion strains were responsible for the M1 and V2 transmission pattern.

**FIG 2:**
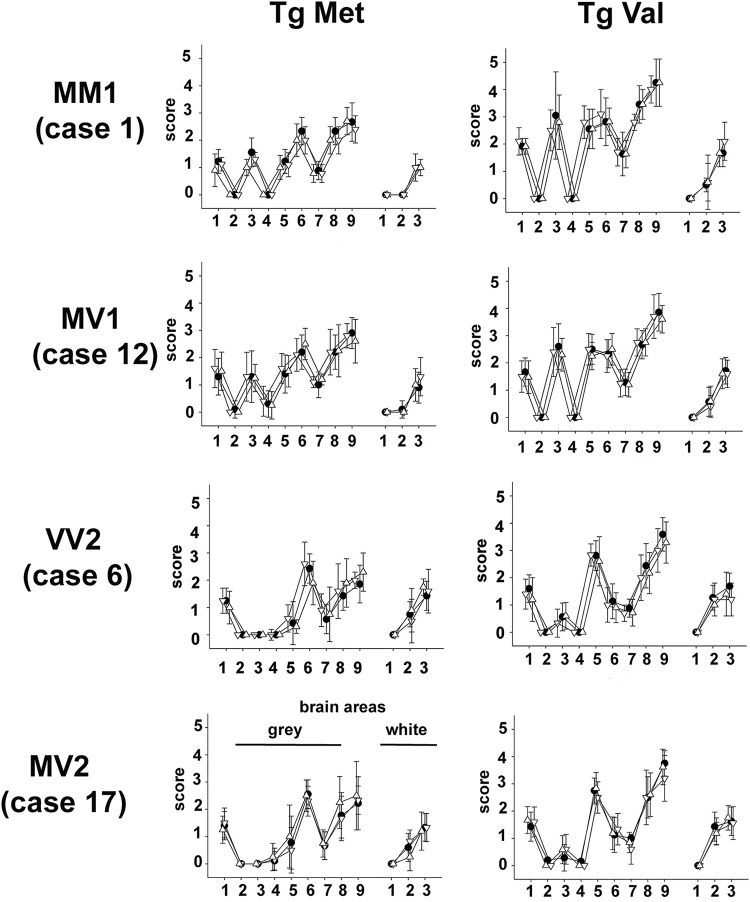
Vacuolar lesion profiles in the brains of human-PrP-expressing mice (tgHu) inoculated with tgHu-adapted sCJD isolates. Transgenic mice that express the Met_129_ (tgMet), Val_129_ (tgVal) human PrP were inoculated intracerebrally (6 mice, 20 μl per mouse) with a 10% brain homogenate (frontal cortex) from one MM1 sCJD patient (case 1), one MV1 sCJD patient (case 12), one VV2 sCJD patient (case 6), and one MV2 sCJD patient (case 17). Two iterative passages were performed for each line (Table 2). For each original sCJD isolate, the prions that were obtained after the two passages in tgMet and tgVal were transmitted (third passage) in both tgMet and tgVal. Graphs present the standardized vacuolar lesion profiles in the brains of the mice inoculated with the original sCJD isolates (•) and the prions obtained after the second passage in tgMet (▵) and tgVal (▽).

To confirm this, prions that propagated in tgMet and tgVal mice inoculated with sCJD isolates 1, 6, 12, and 20 (2nd passage) were reinoculated in tgVal and tgMet mice, respectively. The transmission patterns (incubation periods and PrP^res^ Western blot type [Table 2]) and the brain vacuolar lesion profiles that were observed in tgMet and tgVal mice were identical to those obtained when the original isolates were inoculated in the same mouse lines (Fig. 2). These results demonstrated that the M1^CJD^ and V2^CJD^ transmission patterns in tgMet and tgVal mice were specific for two distinct and apparently stable sCJD prion strains.

**TABLE 2:** Transmission of sporadic CJD isolates adapted (two iterative passages) in mice expressing the methionine 129 or valine 129 human PrP variant^*a*^

Case	Codon 129	PrP^res^ type	Origin	tgMet_129_			tgVal_129_		
				D/I^*b*^	IncubP^*c*^	PrP^res^ type	D/I	IncubP	PrP^res^ type
1	MM	1	2nd pass tgMet	6/6	202 ± 11	1	6/6	298 ± 9	1
			2nd pass tgVal	6/6	212 ± 5	1	6/6	276 ± 5	1

12	MV	1	2nd pass tgMet	6/6	197 ± 3	1	6/6	286 ± 3	1
			2nd pass tgVal	6/6	214 ± 9	1	6/6	270 ± 8	1

6	VV	2	2nd pass tgMet	5/5	508 ± 22		6/6	182 ± 7	2
			2nd pass tgVal	6/6	498 ± 34	1	6/6	171 ± 3	2

20	MV	2	2nd pass tgMet	6/6	483 ± 44	1	6/6	181 ± 4	2
			2nd pass tgVal	5/5	503 ± 27	1	6/6	169 ± 7	2

a Four sCJD cases displaying different *PRNP* genotypes at codon 129 (MM, homozygous Met_129_; VV, homozygous Val_129_; MV, heterozygous Met/Val_129_) and PrP^res^ Western blot isoform (type 1 or type 2) were selected. The prions obtained after two passages (2nd pass) of these isolates in transgenic mice that express the Met_129_ (tgMet) or Val_129_ (tgVal) human PrP were reinoculated (third passage) into tgVal and tgMet (5 or 6 mice; 20 μl per mouse). Brains from second passage mice were pooled and used as inoculum for the third passage. The PrP^res^ WB isoforms (type 1 or type 2) identified in mouse brains are reported.

b D/I, number of diseased mice/number of inoculated mice.

c IncubP, incubation period (time to death in days) shown as means ± standard deviations.

### Discrepant transmission profile.

**FIG 3:**
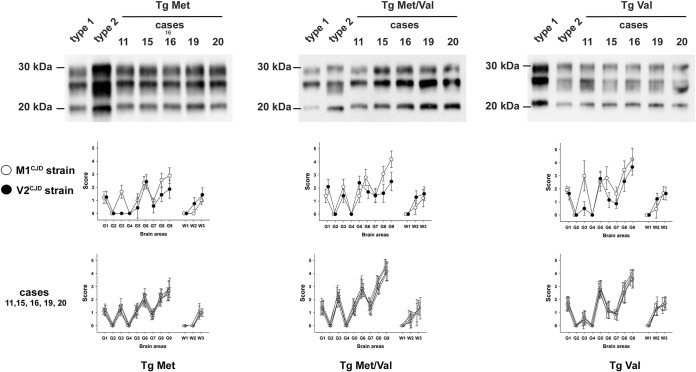
PrP^res^ Western blot profiles and vacuolar lesion profiles in the brains of human PrP-expressing mice (tgHu) inoculated with discrepant phenotype sCJD cases. VV2 (case 11 [▵]), MV1 (case 15 [▽] and case 16 [□]) and MV2 (case 19 [○] and case 20 [◊]) sCJD isolates were inoculated into transgenic mice that express Met_129_ (tgMet), Val_129_ (tgVal) human PrP and their crossbreed (tgMet/Val) (intracerebral route, 6 mice, 20 μl per mouse). After two iterative passages in tgMet, tgVal, and tgMet/Val mice, the PrP^res^ WB profile was established in the mouse brains by SDS-PAGE and WB with the anti-PrP monoclonal antibody Sha31 (epitope YEDRYYRE). A PrP^res^ type 1 isoform (MM1 sCJD isolate [case 1]) and type 2 isoform (VV2 sCJD isolate [case 2]) were included as controls on each gel. In parallel, standardized vacuolar lesion profiles in the brains of the mice that were inoculated with each isolate were established. Lesion profiles corresponding to M1^CJD^ (○) and V2^CJD^ (•) strains are presented for comparison.

The transmission in tgHu mice of 5 out of 20 isolates that correspond to patients who displayed a single PrP^res^ Western blot signature in their brain (Table 1) resulted in a phenotype that differed from M1^CJD^ and V2^CJD^. This “discrepant” transmission pattern was observed in some of the VV2 (case 11), MV2 (cases 15 and 16), and MV1 (cases 19 and 20) isolates. It was characterized by a short incubation period in tgMet mice (about 200 dpi), an intermediate incubation period in tgMet/Val mice (about 250 dpi), and a short incubation period in tgVal mice (about 180 dpi) (Table 1). A type 1 PrP^res^ accumulated in the brains of tgMet and tg Met/Val mice, while type 2 PrP^res^ was observed in tgVal mice (Fig. 3). In these discrepant isolates, the vacuolar lesion profiles in the tgMet and tgMet/Val mice matched the profiles observed in mice inoculated with the M1^CJD^ strain, while in the tgVal mice, the profile matched the profile for mice inoculated with the V2^CJD^ strain (Fig. 3).

**FIG 4:**
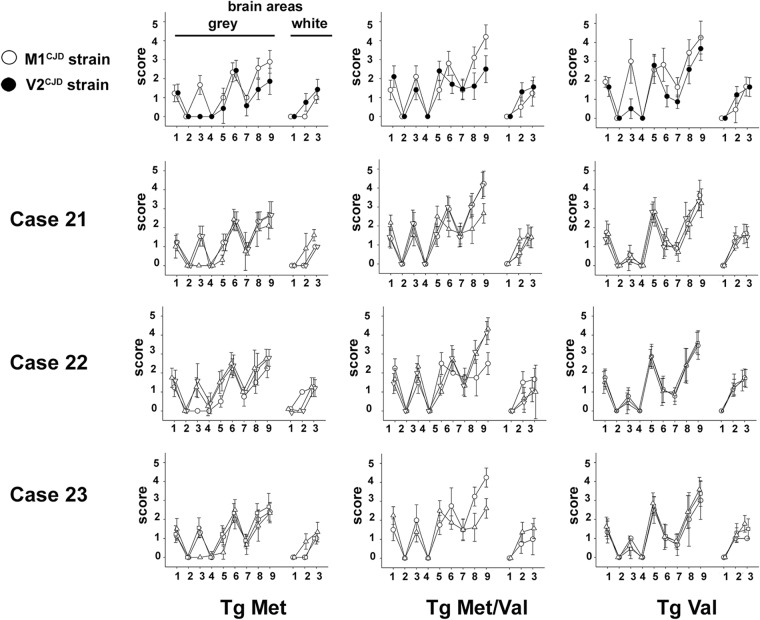
Vacuolar lesion profiles in the brains of human PrP-expressing mice (tgHu) inoculated with sCJD cases displaying mixed type 1/type 2 PrP^res^ WB profiles. Different brain area samples collected from VV (case 21) and MV (cases 22 and 23) sCJD patients displaying both type 1 and type 2 PrP^res^ WB profile in their brain were inoculated (two iterative passages) in transgenic mice that express Met_129_ (tgMet) or Val_129_ (tgVal) human PrP and their crossbreed (tgMet/Val) (intracerebral route, 6 mice, 20 μl per mouse). After two passages, standardized vacuolar lesion profiles in the brains of the mice inoculated with the caudate nucleus (○), cerebellum (▵), temporal cortex (▽), or occipital cortex (□) from the different sCJD cases were established. Lesion profiles corresponding to M1^CJD^ (○) and V2^CJD^ (•) strains in the same tgHu lines are presented for comparison.

Strikingly, inoculation of the 9 isolates that corresponded to the three sCJD patients who displayed a mixed type 1/type 2 PrP^res^ Western blot profile resulted in two distinct phenotypes (incubation period, PrP^res^ Western blot pattern, and vacuolar lesion profile). Seven of these isolates displayed a phenotype identical to those observed for the discrepant isolates, while two others (cases 21 and 23, cerebellum) displayed a V2 strain phenotype (Table 3 and Fig. 4).

**TABLE 3:** Transmission of sporadic CJD isolates displaying complex PrP^res^ profile in mice expressing the human PrP either the methionine or the valine at codon 129^*a*^

Isolate				tgMet						tgMet/Val						tgVal					
				Passage 1			Passage 2			Passage 1			Passage 2			Passage 1			Passage 2		
Case	Codon 129	PrP^res^ type	Brain area^*b*^	D/I^*c*^	IncubP^*d*^	PrP^res^ type	D/I	IncubP	PrP^res^ type	D/I	IncubP	PrP^res^ type	D/I	IncubP	PrP^res^ type	D/I	IncubP	PrP^res^ type	D/I	IncubP	PrP^res^ type
21	VV	1	Caudate	6/6	346 ± 75	1	6/6	215 ± 12	1	6/6	369 ± 52	1	6/6	245 ± 8	1	6/6	196 ± 7	2	6/6	174 ± 7	2
		2	Cerebellum	5/5	569 ± 89	1	6/6	504 ± 74	1	6/6	483 ± 23	1	6/6	504 ± 54	1	6/6	184 ± 11	2	6/6	170 ± 10	2
		2	Temp. Cort	6/6	333 ± 61	1	6/6	221 ± 8	1	6/6	385 ± 61	1	6/6	241 ± 10	1	6/6	208 ± 8	2	6/6	170 ± 9	2

22	MV	2	Caudate	6/6	518 ± 84	1	6/6	571 ± 14	1	6/6	466 ± 90	1	5/5	476 ± 26	1	6/6	180 ± 8	2	6/6	161 ± 8	2
		1+2	Cerebellum	6/6	357 ± 38	1	6/6	220 ± 5	1	6/6	396 ± 66	1	6/6	261 ± 11	1	6/6	180 ± 4	2	6/6	175 ± 5	2
		2	Occip. Cort	6/6	277 ± 50	1	6/6	210 ± 5	1	6/6	387 ± 17	1	6/6	245 ± 21	1	6/6	217 ± 23	2	6/6	164 ± 7	2

23	MV	1+2	Caudate	5/6	519 ± 88	1	6/6	212 ± 8	1	6/6	523 ± 82	1	6/6	250 ± 2	1	6/6	251 ± 66	2	6/6	168 ± 3	2
		2	Cerebellum	0/5	>750*	1	6/6	579 ± 20	1	0/6	>750*	1	6/6	484 ± 40	1	6/6	316 ± 52	2	6/6	166 ± 4	2
		1	Temp. Cort	1/6	689, 718*	1	6/6	254 ± 30	1	0/6	>750	−^*e*^		ND		6/6	362 ± 58	1	6/6	193 ± 13	2

a Three sCJD cases displaying different *PRNP* genotypes at codon 129 (VV, homozygous Val_129_; MV, heterozygous Met/Val_129_) and showing mixed type 1/type 2 PrP^res^ Western blot profiles in either the same or different brain areas were selected. Three brain areas from each case were inoculated (two iterative passages) in transgenic mice that express the Met_129_ (tgMet) or Val_129_ (tgVal) human PrP and their crossbreed (tgMet/Val) were inoculated intracerebrally (20 μl per mouse). Brains from first passage mice (PrP^res^ positive in the brain) were pooled and used for the second passage. The PrP^res^ WB isoforms (type 1 or type 2) identified in mouse brains are reported.

b Abbreviations: Caudate, caudate nucleus; Temp. Cort, temporal cortex; Occip. Cort, occipital cortex.

c D/I, number of diseased mice/number of inoculated mice. *, PrP^res^ positive in a found dead animal without TSE clinical signs.

d IncubP, incubation period (time to death [in days]) shown as means ± standard deviations. An asterisk (e.g., >750*) indicates that all mice were PrP^res^ positive, but showed no clinical signs when killed (end of their life span). ND, not done.

e −, PrP^res^ negative.

These observations led us to consider the hypothesis that the discrepant phenotype might result from the presence of both M1 and V2 strains in the isolates.

### Artificial M1/V2 mixtures reproduce the discrepant phenotype.

In order to test our discrepant phenotype hypothesis, artificial M1^CJD^/V2^CJD^ strain mixtures were created. M1^CJD^ and V2^CJD^ strains were obtained by biological cloning (endpoint titration) of an MM1 isolate (case 1) and a VV2 isolate (case 6) in tgMet and tgVal mice, respectively (see Table S1 in the supplemental material). Strain mixtures were prepared as 1/10 dilution series of a reference M1^CJD^ strain stock homogenate (10% tgMet brain homogenate) in a reference V2^CJD^ strain stock homogenate (10% tgVal brain homogenate) or vice versa. These artificial strain mixtures were then inoculated (two iterative passages) into tgMet and tgVal mice (Table 4).

**TABLE 4:** Bioassay transmission in tgHu mice and PMCA seeding activity detection limit of artificial V2^CJD^/M1^CJD^ strain mixture^*a*^

Artificial strain mixture composition		tgMet_129_						tgVal_129_					
		Passage 1			Passage 2			Passage 1			Passage 2		
		D/I^*b*^	IncubP^*c*^	PrP^res^ type	D/I	IncubP	PrP^res^ type	D/I	IncubP	PrP^res^ type	D/I	IncubP	PrP^res^ type
V2 neat	M1 neat	6/6	207 ± 3	1	6/6	205 ± 2	1	6/6	173 ± 7	2	6/6	179 ± 1	2
V2 neat		6/6	549 ± 65	1	6/6	506 ± 17	1	6/6	175 ± 5	2	6/6	174 ± 3	2
M1 neat		6/6	210 ± 12	1	6/6	199 ± 3	1	6/6	295 ± 5	1	6/6	283 ± 11	1

V2 neat	M1 10^−1^	6/6	244 ± 11	1	6/6	203 ± 8	1	6/6	177 ± 6	2		ND	
	M1 10^−2^	6/6	250 ± 5	1	6/6	198 ± 3	1	6/6	171 ± 6	2		ND	
	M1 10^−3^	6/6	329 ± 89	1	6/6	207 ± 5	1	6/6	173 ± 10	2		ND	
	M1 10^−4^	6/6	463 ± 67	1	6/6	201 ± 7	1	6/6	176 ± 10	2		ND	
	M1 10^-5^	6/6	513 ± 32	1	6/6	505 ± 24	1	6/6	176 ± 5	2		ND	

M1 neat	V2 10^-1^	6/6	207 ± 10	1		ND		6/6	190 ± 8	2	6/6	179 ± 3	2
	V2 10^-2^	6/6	211 ± 9	1		ND		6/6	233 ± 13	2	6/6	189 ± 8	2
	V2 10^-3^	6/6	199 ± 9	1		ND		6/6	245 ± 2	2	6/6	189 ± 8	2

M1 neat	V2 10^-4^	6/6	203 ± 8	1		ND		6/6	288 ± 2	1	6/6	229*^d^	2
												266, 267, 287, 288, 299	1

M1 neat	V2 10^-5^	6/6	202 ± 8	1		ND		6/6	283 ± 8	1	6/6	289 ± 5	1

a M1^CJD^ and V2^CJD^ strains were obtained by the endpoint titration of an MM1 (case 1) and VV2 (case 6) sCJD isolate in Met_129_ (tgMet) or Val_129_ (tgVal) human PrP-expressing mice, respectively (see Table S1 in the supplemental material). Brains from tgMet (inoculated with M1^CJD^ strain) and tgVal (inoculated with V2^CJD^ strain) were used to produce stock solutions (10% tissue homogenates). A dilution series (1/10 dilution series) (in phosphate-buffered saline) of each stock solution was prepared. M1^CJD^/V2^CJD^ strain mixtures were obtained by mixing an equal volume of each component at the chosen dilutions. Samples were then transmitted (two iterative passages) to tgMet and tgVal mice (intracerebral route, 20 μl per mouse). Brains from first-passage-positive mice (PrP^res^ presence in the brain) were pooled and used for the second passage. The PrP^res^ WB isoforms (type 1 or type 2) identified in mouse brains are reported.

b D/I, number of diseased mice/number of inoculated mice. ND, not done.

c IncubP, incubation period (time to death [in days]). The incubation periods are shown as means ± standard deviations.

d The asterisk indicates that the PrP^res^ WB profile of the mice in this group were not homogenous, and the individual incubation period of each animal is presented.

10.1128/mBio.00393-20.1 TABLE S1 Endpoint titration of sporadic CJD MM1 (case 1) and VV2 (case 10) isolates in transgenic mice expressing the human PrP. Successive 1/10 dilutions of 10% brain homogenate (frontal cortex) from an MM1 (case 1) and a VV2 (case 2) sCJD-affected patient were inoculated intracerebrally into tgMet mice (*n *= 6) and tgVal mice (*n *= 6). Mice were euthanized when they showed clinical signs of infection or after 650 days. The mice were considered infected when PrP^res^ deposition was detected in their brain by Western blotting using the Sha31 monoclonal antibody, which recognizes amino acids 145 to 152 (YEDRYYRE) of the sheep PrP. ND, not done; n/n0, number of diseased mice/number of inoculated mice. Incubation periods (time to death [in days]) are shown as means ± standard deviations (SD) except when less than 100% of the animals developed clinical signs. In that case, individual incubation period are presented. The data included in this table were already used in Huor et al. (49). Download Table S1, DOCX file, 0.02 MB. Copyright © 2020 Cassard et al. 2020 Cassard et al. This content is distributed under the terms of the Creative Commons Attribution 4.0 International license.

In tgVal mice, the V2 strain phenotype (160 to 180 dpi, type 2 PrP^res^ Western blot profile) was observed, after the first and/or second passage, in mice that received M1^CJD^/V2^CJD^ strain mixtures containing more than 10^−3^ diluted V2^CJD^ strain. Below this level, the M1^CJD^ strain phenotype was observed in tgVal mice. In tgMet mice, the M1^CJD^ strain phenotype (190 to 210 dpi, type 1 PrP^res^ Western blot profile) was recovered after the first and/or second passage in mice that received strain mixtures more than a 10^−4^ diluted M1^CJD^ strain. Below this level, the V2^CJD^ strain phenotype was observed in the inoculated tgMet mice.

These results demonstrate that the discrepant phenotype observed when transmitting certain sCJD isolates in tgMet and tgVal mice can be recreated by *in vitro* mixing M1^CJD^/V2^CJD^ strains. The data also indicate that the presence of a relatively low level of M1^CJD^ and V2^CJD^ prion strains in isolates dominated by the V2^CJD^ and M1^CJD^ strains, respectively, is sufficient to result in a discrepant transmission phenotype in tgHu mice.

### M1^CJD^/V2^CJD^ strain mixture in sCJD patients.

In order to provide direct evidence for the existence of M1^CJD^/V2^CJD^ strain mixtures in the brains of sCJD patients, we established a protein misfolding cyclic amplification (PMCA) protocol that specifically identifies the seeding activity of the V2^CJD^ prion strain. PMCA is a methodology that mimics prion replication *in vitro*, but in an accelerated form, allowing amplification of minute amounts of PrP^Sc^ and prion infectivity (16). In PMCA, a PrP^C^-containing “substrate” is combined with a “seed” that may contain otherwise undetectable amounts of PrP^Sc^. After repeated cycles of incubation and sonication, the amount of PrP^Sc^ increases to levels where they can be detected by conventional biochemical methods.

**FIG 5:**
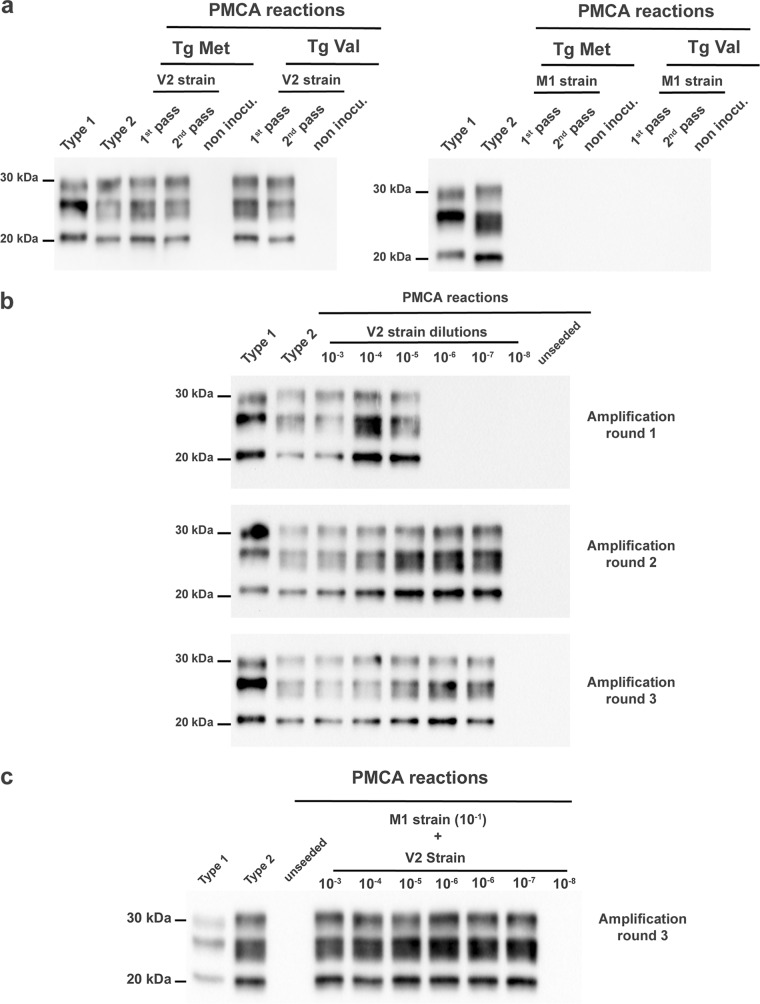
Amplification of M1^CJD^ and V2^CJD^ strains by protein misfolding cyclic amplification (PMCA). (a) PMCA reactions were seeded with brain homogenate from methionine 129 (tgMet) and valine 129 (tgVal) mice inoculated (1st and 2nd passage) with M1^CJD^ and V2^CJD^ prion strains (10^−2^ dilution of a 10% brain homogenate). The PMCA substrate was prepared using tgVal brains. Unseeded reactions were included as specificity controls. PMCA reactions were then subjected to 6 rounds of amplification, each comprising 96 cycles (10-s sonication for 14 min and 50-s incubation at 39.5°C) in a Qsonica700. After each PMCA round, reaction products (1 volume) were mixed with fresh substrate (9 volumes) to seed the following round. After six PMCA rounds, the products were analyzed by Western blotting (WB) for the presence of abnormal PK-resistant PrP (PrP^res^ antibody Sha31 epitope YEDRYYRE). A type 1 (MM1 [case 1]) and type 2 (VV2 [case 6]) PrP^res^ controls were included as control on each WB gel. (b) PMCA reactions were seeded with 10-fold serial dilutions of an V2^CJD^ strain (tgVal 10% brain homogenate, 10^−3^ to 10^−8^ dilution in PMCA buffer). Six rounds of amplification were performed. After each round, a part of the product was analyzed by WB for the presence of PrP^res^. (c) PMCA reactions were seeded with 10-fold serial dilutions of a V2^CJD^ strain (tgVal 10% brain homogenate, 10^−3^ to 10^−8^ dilution) in an M1^CJD^ strain (10^−1^ dilution of a 10% tgMet brain homogenate).

Biologically cloned M1^CJD^ and V2^CJD^ prion strains were obtained by endpoint titration of sCJD isolates in tgMet and tgVal mice (Table S1). PMCA reactions that were seeded with V2^CJD^ cloned prions passaged in tgMet or tgVal mice were positive and displayed a type 2 PrP^res^ Western blot profile. Conversely, reactions seeded with M1^CJD^ cloned prions passaged in tgMet or tgVal mice were PrP^res^ negative on Western blot (Fig. 5a). This indicated that the PMCA protocol was able to selectively amplify the V2^CJD^ strain, but not the M1^CJD^ strain.

To determine the sensitivity of V2^CJD^ detection by this PMCA, the reference V2^CJD^ strain material that had previously undergone endpoint titration in tgVal mice (intracerebral [i.c.] inoculation route) (Table S2) was retitrated by PMCA (Fig. 5b). The amplification of a 10-fold serial dilution of this sample (6 individual replicates per dilution point) demonstrated that three PMCA rounds (24 h per round, i.e., 72 h) were sufficient to reach the maximal sensitivity level of the assay. Additional PMCA rounds improved neither the analytical sensitivity of the assay nor the number of positive replicates (Fig. 5b). Importantly, the presence of the M1^CJD^ strain did not affect the sensitivity of V2^CJD^ strain detection (Fig. 5c).

10.1128/mBio.00393-20.2 TABLE S2 Endpoint titration of biologically cloned V2^CJD^ strain isolate in transgenic mice expressing the human PrP (valine 129 variant) and by protein misfolding cyclic amplification. Successive 1/10 dilutions of 10% brain homogenate of tgVal mice inoculated with a V2^CJD^ strain (obtained by endpoint titration of VV2 sCJD case 6 in tgVal mice) was prepared in phosphate-buffered saline. The dilution series was inoculated intracerebrally into tgVal mice (*n *= 6). Mice were euthanized when they showed clinical signs of infection or after 650 days. The mice were considered infected when PrP^res^ deposition was detected in their brain by Western bloting using the Sha31 monoclonal antibody, which recognizes amino acids 145 to 152 (YEDRYYRE) of the sheep PrP. The same dilution series was used to seed PMCA reactions (6 replicates per dilution). After three PMCA rounds, amplification products were analyzed by Western blotting (WB) for the presence of abnormal PK-resistant PrP (PrP^res^ antibody Sha31 epitope YEDRYYRE). The number of PrP^res^ WB-positive replicates corresponding to each round and each dilution are reported. Infectivity titer (50% infectious dose [ID_50_] per ml of 10% brain homogenate) and 50% seeding activity titer (SA_50_ per ml of 10% brain homogenate) were estimated using Spearman Karber’s limiting dilution titration method (most likely value). ND, not done. Incubation periods (in days) are shown as means ± standard deviations (SD) except when less than 100% of the animals developed clinical signs. In that case, individual incubation periods are presented. Download Table S2, DOCX file, 0.02 MB. Copyright © 2020 Cassard et al. 2020 Cassard et al. This content is distributed under the terms of the Creative Commons Attribution 4.0 International license.

Based on these results, the seeding activity (SA) of the isolate was estimated by the Spearman-Kärber method to be 10^9.3^ 50% SA (SA_50_) per ml of 10% brain homogenate. The bioassay endpoint titration data of the same sample in tgVal mice gave an infectious titer of 10^5.53^ 50% lethal dose (LD_50_) per ml of tissue homogenate (Table S2). Taking into account the 4-fold-lower amount of material used to seed the PMCA reaction compared to the material used in mouse inoculations, these results indicate that the PMCA protocol we used was ∼1,500 times more sensitive than the bioassay in tgVal mice for the detection of V2^CJD^ prions.

**FIG 6:**
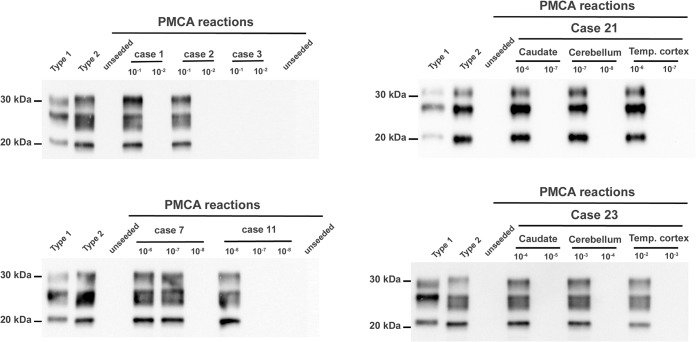
Specific probing of the V2^CJD^ strain in sCJD isolates by protein misfolding cyclic amplification. PMCA reactions were seeded with a 1/10 dilution series (10^−1^ to 10^−8^ dilutions of 10% brain homogenate in PMCA buffer) corresponding to different sCJD patients and/or different brain areas from a single patient (Tables 1 and 3). PMCA reactions were subjected to 3 rounds of amplification, each comprising 96 cycles (10-s sonication for 14 min and 50-s incubation at 39.5°C) in a Qsonica700. This PMCA protocol allows amplification of the V2^CJD^ strain, but not the M1^CJD^ strain (Fig. 5). After three PMCA rounds, products were analyzed by Western blotting (WB) for the presence of abnormal PK-resistant PrP (PrP^res^ antibody Sha31 epitope YEDRYYRE). A type 1 (case 1) and type 2 (case 6) PrP^res^ were included as controls on each WB gel. For each sample, the WB shows only PrP^res^ presence/absence at dilutions bracketing the endpoint dilutions (Table 5).

Finally, the presence/level of V2^CJD^ prion seeding activity in sCJD isolates originally characterized by bioassay in tgHu mice was assessed by PMCA. A 1/10 dilution series of the samples originally used to inoculate mice was prepared and used to seed PMCA reactions (6 PMCA reactions per dilution). After three PMCA amplification rounds, reaction products were tested for the presence of PrP^res^ by Western blotting (Fig. 6), and prion seeding activity titers were estimated using the Spearman-Kärber method, or, when appropriate, Poisson’s probabilistic model (Table 5).

**TABLE 5:** V2^CJD^ strain seeding activity endpoint titration in sCJD isolates^*a*^

Isolate				Strain typing in tgHu mice	No. of mice showing V2 strain PMCA amplification/total no. of mice tested^*c*^								V2 strain seeding activity (SA_50_/ml)^*d*^
Case	Codon 129	PrP^res^ type	Brain area^*b*^		10^−1^	10^−2^	10^−3^	10^−4^	10^−5^	10^−6^	10^−7^	10^−8^	
1	MM	1	Front Cort	M1	**4/6**	0/6	0/6						10^2.34^
2			Front Cort	M1	**2/6**	0/6	0/6						10^1.91^
3			Front Cort	M1	0/6	0/6	0/6						
4			Front Cort	M1	**1/6**	0/6	0/6						10^1.56^

6	VV	2	Front Cort	V2	**6/6**	**6/6**	**6/6**	**6/6**	**6/6**	**6/6**	**4/6**	0/6	10^9.47^
8			Front Cort	V2	**6/6**	**6/6**	**6/6**	**6/6**	**3/6**	0/6	0/6	0/6	10^7.3^
11			Front Cort	M1+V2	**6/6**	**6/6**	**6/6**	**6/6**	**5/6**	**2/6**	0/6	0/6	10^7.97^

12	MV	1	Front Cort	M1	0/6	0/6	0/6	0/6					
13			Front Cort	M1	0/6	0/6	0/6	0/6					
14			Front Cort	M1	0/6	0/6	0/6	0/6					
15			Front Cort	M1+V2	**6/6**	**6/6**	**3/6**	0/6	0/6	0/6			10^4.3^
16			Front Cort	M1+V2	**6/6**	**6/6**	**4/6**	**1/6**	0/6	0/6			10^4.63^

17	MV	2	Front Cort	V2	**6/6**	**6/6**	**6/6**	**6/6**	**4/6**	**1/6**	0/6	0/6	10^7.63^
20			Front Cort	M1+V2	**6/6**	**6/6**	**6/6**	**6/6**	**6/6**	**5/6**	**1/6**	0/6	10^8.8^

21	VV	1	Caudate	M1+V2	**6/6**	**6/6**	**6/6**	**6/6**	**6/6**	**4/6**	0/6	0/6	10^8.47^
		2	Cerebellum	V2	**6/6**	**6/6**	**6/6**	**6/6**	**6/6**	**5/6**	**3/6**	0/6	10^9.13^
		2	Temp. Cort	M1+V2	**6/6**	**6/6**	**6/6**	**6/6**	**4/6**	**1/6**	0/6	0/6	10^7.63^

22	MV	2	Caudate	V2	**6/6**	**6/6**	**6/6**	**6/6**	**6/6**	**5/6**	**1/6**	0/6	10^8.8^
		1+2	Cerebellum	M1+V2	**6/6**	**6/6**	**6/6**	**6/6**	**6/6**	**3/6**	**2/6**	0/6	10^8.63^
		2	Occip. Cort	M1+V2	**6/6**	**6/6**	**6/6**	**6/6**	**6/6**	**2/6**	0/6	0/6	10^8.13^

23	MV	1+2	Caudate	M1+V2	**6/6**	**6/6**	**4/6**	**2/6**	0/6	0/6			10^4.8^
		2	Cerebellum	V2	**6/6**	**6/6**	**3/6**	0/6	0/6	0/6			10^4.3^
		1	Temp. Cort	M1+V2	**6/6**	**4/6**	**1/6**	0/6	0/6	0/6			10^3.63^

a PMCA reactions were seeded with a 1/10 dilution series of isolates (10^−1^ to 10^−8^ dilutions of 10% brain homogenate) corresponding to different sCJD affected patients and/or different brain areas from the same patient (see Tables 1 and 3). The sample aliquots used to prepare dilution series were identical to those used for transmission experiments in mice (Tables 1 and 3). For each sample and each dilution, six individual PMCA reactions were seeded. The PMCA protocol allows amplifying the V2^CJD^ strain, but not the M1^CJD^ strain (Fig. 5). After three PMCA rounds, products were analyzed by Western blotting (WB) for the presence of abnormal PK-resistant PrP (PrP^res^ antibody Sha31 epitope YEDRYYRE). The number of PrP^res^ WB-positive replicates corresponding to each round and each dilution are reported.

b Abbreviations: Front Cort, frontal cortex; Caudate, caudate nucleus; Temp. Cort, temporal cortex; Occip. Cort, occipital cortex.

c Values for which one or more mice of the six mice tested showed V2 strain PMCA amplication are shown in boldface type.

d Seeding activity titers were estimated using Spearman Karber’s limiting dilution titration method (most likely value) or (when less than 100% positive reaction was observed in the first tested dilution) by the Poisson’s probabilistic model as described by Brown et al. (51). Titers are given as the number of PMCA 50% seeding activity (SA_50_) per milliliter of 10% brain homogenate.

A high seeding activity (>10^7^ SA_50_/ml) was observed in all the sCJD isolates that displayed a V2^CJD^ profile in bioassay. Moderate (>10^3.6^ SA_50_/ml) to very high (>10^8^ SA_50_/ml) seeding activity were observed in the isolates that displayed a discrepant transmission phenotype in tgHu mice. Unexpectedly, low but consistent V2^CJD^ seeding activity were also observed in 3 out 4 MM1 sCJD isolates that displayed a pure M1^CJD^ transmission profile in tgHu mice. However, the seeding activity titers in these particular isolates were largely below the ∼10^3.8^ SA_50_/ml that corresponded to the estimated detection limit of the V2 ^CJD^ prion by bioassay in tgVal mice (Table 5). PrP^res^-positive reactions all displayed a Western blot profile that was similar to the profile of the original V2^CJD^ prion strain.

## DISCUSSION

The transmission of sCJD (from 23 sCJD cases) to mice that overexpress the methionine and/or valine 129 variants of human PrP^C^ allowed the identification of two dominant sCJD strains, named M1^CJD^ and V2^CJD^, which were preferentially associated with MM/MV1 and MV/VV2 prion disease cases, respectively. These results confirm and expand the observations reported by Bishop et al. on the basis of the inoculation of a limited number of sCJD isolates in knock-in mice that expressed methionine and/or valine 129 human PrP variants (12). Our results are also in agreement with transmission results obtained more recently by other groups using Met_129_ human PrP-overexpressing mice (17–19).

Fifteen years ago, the detection by Western blotting of both type 1 and type 2 PrP^res^ in the brains of a significant number of sCJD patients led to the proposal that more than a single prion strain might be present in these individuals (13, 14). The results of our study presented here definitively demonstrate the existence of M1^CJD^ and V2^CJD^ sCJD prion strain mixtures in MV and VV cases that display both type 1 and type 2 PrP^res^ by Western blotting. However, our study also revealed that M1^CJD^/V2^CJD^ strain mixtures occur in a large proportion of the MM/MV1 and VV/MV2 sCJD cases (more than 40% of the isolates in our panel), including those patients where neither the neuropathological phenotype nor the PrP^res^ Western blot profile suggested its existence.

The copropagation of two prion strains in the brains of sCJD patients and the apparently frequent occurrence of this phenomenon naturally lead to the question of its origin. In the absence of identifiable external sources that might explain sCJD occurrence, it is currently assumed that this disease is triggered by the spontaneous and stochastic formation of a misfolded PrP nucleus in the brain of the affected individual. This original nucleus is considered to recruit and convert nascent PrP^C^ into PrP^Sc^, leading to the progressive spread and propagation of prions in the brain of the diseased patient (20).

Based on the postulate that the conformation of PrP^Sc^ molecules/aggregates encodes prion strain information (21, 22), several mechanisms could explain the cooccurrence of both M1^CJD^ and V2^CJD^ strains in MM/MV1 and MM/VV2 sCJD patients. First, the two prion strains might arise concomitantly during the event(s) responsible for the generation of the initial PrP^Sc^ nucleus. Since M1^CJD^ and V2^CJD^ display different capacities to replicate in mice that express either Met_129_ or Val_129_ human PrP (see Table S1 in the supplemental material), the relative proportion of M1^CJD^ and V2^CJD^ prion strains in the brains of affected individuals might be expected to vary according to their *PRNP* codon 129 genotype. Alternatively, the occurrence of M1^CJD^/V2^CJD^ prion mixtures in sCJD patients could result from the nonclonal replication of prions as proposed by the conformational selection hypothesis (21). This concept proposes that a prion strain naturally propagates in its host as an ensemble of PrP^Sc^ conformers. The ensemble is considered to be dominated by a major PrP^Sc^ conformer that preferentially propagates in the host and is responsible for the observable prion strain phenotype. However, other prion substrains, those associated with minor populations of PrP^Sc^ conformers, are also proposed to be present and emerge as the dominant strain upon transmission to a different host (21, 23). Differences in PrP^C^ posttranslational modifications, metabolism, or expression levels have been shown *in vitro* and *in vivo* to impact the capacity and efficacy of prion strain propagation (24). Variations both within and between different brain areas could exert a selection pressure on the different prion substrains generated or selected during the replication process (25–27). In such a scenario, the combination of host *PRNP* codon 129 genotype with the temporal and topographical region of the brain area(s) where the divergence of the prion strains could occur would determine the final levels of M1^CJD^ and V2^CJD^ prion strains observed in specific brain areas of each patient.

Our study here has investigated a substantial number of sCJD isolates (*n* = 29), which is significantly greater than previous studies aimed at describing the diversity of sCJD prion strains (12, 17–19).

As a consequence, the data we have generated have been instrumental in demonstrating the occurrence of the M1^CJD^/V2^CJD^ strain mixture phenomenon in the MM/MV1 and MV/VV2 sCJD patients. Since our study was focused mainly on the existence of multiple prion strains in specific subgroups of sCJD isolates, we have not been able to fully address the total repertoire of prion strain diversity associated with this particular human prion disease. However, our data indicate that MV2C and MV2K cases (Table 1), which were proposed to be caused by different sCJD strains on the basis of clinical and neuropathological features in patients (5), could be due to the same prion strain. These findings suggest that the same prions could be associated with different forms of prion disease in humans that share the same *PRNP* genotype at codon 129. Alternatively, it could signify that bioassay of these isolates in tgHu mice was unable to reflect their true prion strain diversity.

To date, the existence of a total of five distinct sCJD prion strains have been formally established by bioassay in tgHu mice. In addition to the M1^CJD^ and V2^CJD^ strains identified here, the existence of M2^CJD^ and V1^CJD^ strains was established on the basis of the absence of transmission in knock-in human PrP mice of one MM2C case and one VV1 sCJD case, respectively (12). MM2C was later shown to be transmissible to mice that overexpress human PrP (17). An additional prion strain was identified on the basis of the transmission of one MM2 thalamic case in Met_129_ human PrP mice (28). Considering the relative variability in terms of prion strain content that we observed within the MM/MV1 and VV/MV2 isolates assessed here, it is difficult to reconcile that strain typing by bioassay of a single isolate could provide a reliable reflection of the diversity of prions associated with a particular sCJD clinicopathological phenotype. Moreover, at this time, there is a lack of comprehensive bioassay-based strain typing data for certain clinicopathological subtypes of sCJD, such as the MM2 thalamic forms or MM1 and MM2 cases (5). If mixtures of prion strains other than M1^CJD^/V2^CJD^ exist in sCJD cases, the final characterization of their diversity in this form of human prion disease could be very challenging.

The occurrence and frequency of the sCJD strain mixture phenomenon in sCJD patients will have important implications for the development of therapeutic strategies for prion diseases. The efforts deployed over the last 20 years to identify chemical or biological interventions that would be able to slow or revert the progression of sCJD have remained elusive. The hurdles to surmount the development of therapy against sCJD are numerous (e.g., early diagnosis of CJD in patients, blood-brain barrier passage). The existence of the strain mixture phenomenon reported here could represent an additional difficulty. Indeed, it has been clearly established in different animal and cellular models of prion disease that the efficacy of antiprion compounds can vary significantly according to the prion strain under investigation (23, 29–32). Accordingly, in the context of multiple prion strains within individual sCJD patients, only treatments that are effective against the full spectrum of prion strains are likely to provide a therapeutic benefit.

The results reported in this study demonstrate that two prion strains can be present in the brain of individual sCJD patients. The transmission of variant CJD (vCJD) isolates to human PrP mice was reported to lead to the propagation of either vCJD or sCJD-like prion, suggesting that two prion strains might have been present in the original isolates (33). The coexistence of at least two prion strains in the brain of the same individual was also reported for small ruminants with scrapie, cattle with atypical H-type bovine spongiform encephalopathy (BSE-H) (the presence of minor classical BSE component), and in cervids with chronic wasting disease (34–37). This supports the view that strain mixture occurrence might be a relatively common phenomenon across mammal prion diseases.

The presence in a single individual of two prion strains, including components that remain undetected by standard examination of the isolates, has important consequences for public health. The interactions between the prion strain and host features (mainly defined by the PrP^C^ sequence) determine the distribution and levels of infectivity in the tissues and body fluids of incubating and clinically affected individuals. Moreover, the capacity of a prion to transmit between hosts from different species also depends on the nature of the prion strain (transmission barrier phenomenon) (7). In animals used for human food, the presence of several prion substrains could result in an unrecognized exposure of consumers to minor populations of prions displaying a zoonotic potential.

In humans, iatrogenic transmission has been responsible for the occurrence of several hundreds of CJD cases. These cases have mainly occurred in recipients of human pituitary-derived growth hormone (GH) or human dura mater (DM) grafts (38). The strain mixture phenomenon in patients either incubating or affected by CJD is unlikely to have a major impact on the risks of iatrogenic transmission in exposed patients. However, the coexistence of two or more sCJD strains in donors from whom DM grafts or GH extracts were prepared might have influenced the phenotypic and epidemiological diversity of these iatrogenic cohorts (39–41).

## MATERIALS AND METHODS

### Ethics statement.

All animal experiments were performed in compliance with institutional and French national guidelines in accordance with the European Union directives 86/609/EEC and 2010/63/EU. Experiments were approved by the Committee on the Ethics of Animal Experiments of the authors’ institutions: INRA Toulouse/ENVT (permit number 01734.01) and Instituto Nacional de Investigación y Tecnología Agraria y Alimentaria (INIA) (permit number CEAA 2011/046).

Concerning the human sCJD samples, in all cases, informed consent for research was obtained, and the material used had appropriate ethical approval for use in this project.

In France, human brain samples were obtained from the Brain Bank of CHU of Toulouse (approval number AC62009-973/20-01-2010). Samples were pseudoanonymized before dispatch.

In the United Kingdom (UK), human brain samples were obtained from the National CJD Research & Surveillance Unit Brain and Tissue Bank in Edinburgh, UK, which is part of the MRC Edinburgh Brain Bank. For the purposes of this study, samples were pseudoanonymized using a Brain Bank reference number. All UK cases had informed consent for research, and their supply and use in this study were covered by Ethics Approval (LREC 2000/4/157: National Creutzfeldt-Jakob disease tissue bank: acquisition and use of autopsy material for research on human transmissible spongiform encephalopathies, James Ironside, amended date 9 October 2007).

In Spain, human brain samples were obtained from the Basque Biobank of the Basque Foundation for Health Innovation and Research, Vizcaya, Spain. Samples were anonymized using a Brain Bank reference number. All Spanish cases had informed consent for research, and their supply and use in this study were covered by Ethics Approval of the Euskadi Clinical Research Ethics Committee (CEIC-E 02/2012).

### sCJD cases.

Twenty-three cases of sCJD, each of which had frozen tissue (2 to 4 g) available from 5 brain regions: (occipital, temporal, and frontal cortex, cerebellum, and the caudate nucleus), were included in this study. The patients originated from France, UK, and Spain.

The four major subtypes of sCJD patients (MM1, MV1, MV2, and VV2) were represented in our panel (Table 1 and Table 3). None of the patients had a familial history of prion disease, and in each case, the entire *PRNP* coding sequence was analyzed (42, 43).

### Tissue homogenate preparation.

Frozen brain tissue (175 ± 20 mg) was homogenized in 5% glucose in distilled water in grinding tubes (Bio-Rad) adjusted to 10% (wt/vol) using a TeSeE Precess 48 homogenizer (Bio-Rad).

### Transgenic mouse lines.

tg340 and tg361 mouse lines that express human PrP methionine at codon 129 or valine at codon 129, respectively, in a PrP^Ko^ (Ko stands for knockout) background, were generated as previously described (10, 15). Both tg340 (tgMet) and tg361 (tgVal) are homozygous for the human *PRNP* gene, and tgMet/Val_129_ mice used in our experiments were obtained by mating tg340 and tg361 mice (F1 generation).

### Mouse bioassays.

Six- to ten-week-old female mice were anesthetized and inoculated with 2 μg of brain equivalent (20 μl of a 10% temporal cortex homogenate) in the right parietal lobe using a 25-gauge disposable hypodermic needle.

Mice were observed daily, and their neurological status was assessed weekly by qualified veterinarians. Three signs of neurological dysfunction (any three signs of the following signs: tremor, ataxia, difficulty righting from supine position, rigidity of tail, kyphosis, paralysis of the lower limbs, or bradykinesia) were required to give a mouse a positive score for TSE clinical signs (44).

When clinically progressive TSE disease was evident, the animals were euthanized, and their brains were harvested. Half of the brain from those animals that had displayed TSE clinical signs was fixed by immersion in 10% Formol saline, and the other half was frozen at –20°C. Tissues from mice found dead were frozen (no formalin fixation). The incubation period was expressed as the mean survival time (time to death) (in days postinoculation [dpi]), with its corresponding standard deviation, for the euthanized mice and the mice found dead that were scored positive for both TSE clinical signs and brain PrP^res^. In animals where no clinical signs were observed, mice were killed at the end of their natural life span (750 to 800 days). In cages where no animal developed clinical TSE signs, incubation periods are reported in the table as >650 or >750 dpi.

### Abnormal PrP Western blot detection.

Proteinase K (PK)-resistant abnormal PrP extraction (PrP^res^) and Western blotting (WB) were performed as previously described (45). Immunodetection was performed using the monoclonal PrP-specific antibody Sha31 (1 μg/ml), which recognize the amino acid sequence YEDRYYRE (positions 145 to 152) (46).

### Vacuolar lesion profiles.

Hematoxylin-eosin-stained paraffin-embedded brain tissue sections were used to establish standardized vacuolar lesion profiles in mice as previously described (47, 48). Each lesion profile was based on data obtained from 5 or 6 animals.

### Prion strain biological cloning and endpoint titration.

Successive 1/10 dilutions of 10% brain homogenate (frontal cortex) from a MM1 sCJD patient (case 1) and a VV2 sCJD patient (case 6) were inoculated intracerebrally into tgMet mice (*n* = 6) and tgVal mice (*n* = 6). These data have been presented in a previous study (49). The brains of the last PrP^Sc^-positive MM1 in tgMet mice and VV2 in tgVal mice (in the highest dilution groups) were used to reinoculate groups of tgMet mice (*n* = 12) and tgVal mice (*n* = 12). The brains of these tgMet and tgVal animals were pooled to constitute two reference material stocks (named M1^CJD^ and V2^CJD^). The V2^CJD^ stock homogenate was endpoint titrated by bioassay in tgVal mice (inoculation of successive 1/10 dilutions of 10% homogenates [see Table S2 in the supplemental material]). The V2^CJD^ infectious titer was estimated by the Spearman-Kärber method (50).

### PMCA reactions and prion seeding activity titers.

Brains from tgVal mice were used to prepare the PMCA substrate as previously described (11). PMCA amplification was performed as previously described (45). Each PMCA run included a reference sCJD sample (V2^CJD^ stock homogenate titrated in tgVal mice) as a control for the amplification efficiency. Unseeded controls (1 unseeded control for 8 seeded reactions) were also included in each run. Each dilution of each sample was tested (six replicates) in two independent runs. For each sample, the number of positive replicates (presence of detectable PrP^res^ in the reaction as assessed by WB) is reported. The titer of prion seeding activity (50% seeding activity [SA_50_] per milliliter of homogenate) was estimated by the Spearman-Kärber method (50) or (when less than 100% of the PMCA reactions seeded with the first dilution were found positive) by the limiting dilution titration method (application of Poisson’s probabilistic model) described by Brown et al. (51).
