# Supplementary material for: Prions from Sporadic Creutzfeldt-Jakob Disease Patients Propagate as Strain Mixtures
Source: mBio. 2020 Jun 16;11(3):e00393-20. doi: 10.1128/mBio.00393-20 (PMC7298703; doi:10.1128/mBio.00393-20)
Supplement: TABLE S1 [file mBio.00393-20-st001.docx]

**Sup Table 1:** End point titration of Sporadic CJD MM1 (case 1) and VV2 (case 10) isolates in transgenic mice expressing the human PrP

|  | **MM1 (case 1)** | | | | |  | **VV2 (case 10)** | | | | |
| --- | --- | --- | --- | --- | --- | --- | --- | --- | --- | --- | --- |
|  | **TgMet_129_** | |  | **TgVal_129_** | |  | **TgMet_129_** | |  | **TgVal_129_** | |
|  | **n/n0** | **Incubation period** |  | **n/n0** | **Incubation period** |  | **n/n0** | **Incubation period** |  | **n/n0** | **Incubation period** |
| **neat** | 6/6 | 186±10 |  | 6/6 | 286±18 |  | 6/6 | 585±24 |  | 6/6 | 166±11 |
| **10^-1^** | 6/6 | 213±15 |  | 6/6 | 347±16 |  | ND |  |  | 6/6 | 191±9 |
| **10^-2^** | 6/6 | 240±13 |  | 1/6 | 390 |  | ND | - |  | 6/6 | 203±9 |
| **10^-3^** | 6/6 | 263±24 |  | 0/6 | >650 |  | ND | - |  | 6/6 | 252±16 |
| **10^-4-^** | 6/6 | 296±26 |  | 0/6 | >650 |  | ND | - |  | 1/6 | 294 |
| **10^-5^** | 6/6 | 323±29 |  | 0/6 | >650 |  | ND | - |  | 0/6 | >650 |
| **10^-6^** | 1/6 | 316 |  | 0/6 | >650 |  | ND | - |  | 0/6 | >650 |
| **10^-7^** | 0/6 | >600 |  | ND | - |  | ND | - |  | 0/6 | >650 |

Successive 1/10 dilutions of 10% brain homogenate (frontal cortex) from an MM1(case 1) and a VV2 (case 2) sCJD-affected patient were inoculated intracerebrally to tgMet (*n*=6) and tgVal mice (*n*=6). Mice were euthanized when they showed clinical signs of infection or after 650 days. Mice were considered infected when PrP^res^ deposition was detected in their brain by western blot using the Sha31 monoclonal antibody, which recognizes amino acids 145–152 (YEDRYYRE) of the sheep PrP. ND: not done. n/n0: number of diseased / number of inoculated mice. Incubation periods (time to death in days) are shown as mean±standard deviation (SD) except when less than 100% of the animals developed clinical sign. In that case individual incubation period are presented.The data included in this table were already used in Huor et al 2017 [21].
