# Supplementary material for: Prions from Sporadic Creutzfeldt-Jakob Disease Patients Propagate as Strain Mixtures
Source: mBio. 2020 Jun 16;11(3):e00393-20. doi: 10.1128/mBio.00393-20 (PMC7298703; doi:10.1128/mBio.00393-20)
Supplement: TABLE S2 [file mBio.00393-20-st002.docx]

**Sup Table 2** End point titration of biologically cloned V2^CJD^ strain isolate in transgenic mice expressing the human PrP (Valine 129 variant) and by Protein Misfolding Cyclic Amplification

|  |  | **TgVal_129_** | |  | **PMCA** |
| --- | --- | --- | --- | --- | --- |
|  |  | **positive mice** | **Incubation period** |  | **PrP^res^ positive reactions** |
| **neat** |  | 6/6 | 164±5 |  | ND |
| **10^-1^** |  | 6/6 | ND |  | 6/6 |
| **10^-2^** |  | 6/6 | ND |  | 6/6 |
| **10^-3^** |  | 6/6 | 263±12 |  | 6/6 |
| **10^-4-^** |  | 2/6 | 283, 302 |  | 6/6 |
| **10^-5^** |  | 0/6 | >600 |  | 6/6 |
| **10^-6^** |  | 0/6 | >600 |  | 6/6 |
| **10^-7^** |  | 0/6 | >600 |  | 3/6 |
| **10^-8^** |  | ND |  |  | 0/6 |
| **10^-9^** |  | ND |  |  | 0/6 |
|  |  |  |  |  |  |
| **Titer** |  | 10^5.53^ ID_50_ / mL | |  | 10^9.3^ SA_50_ / mL |

Successive 1/10 dilutions of 10% brain homogenate of tgVal mice inoculated with a V2^CJD^ strain (obtained by end point titration of VV2 sCJD case 6 in tgVal) was prepared in Phosphate buffer saline. The dilution series was inoculated intracerebrally to tgVal mice (*n*=6). Mice were euthanized when they showed clinical signs of infection or after 650 days. Mice were considered infected when PrP^res^ deposition was detected in their brain by western blot using the Sha31 monoclonal antibody, which recognizes amino acids 145–152 (YEDRYYRE) of the sheep PrP. The same dilution series was used to seed PMCA reactions (6 replicates per dilution). After three PMCA rounds, amplification products were analyzed by Western Blot (WB) for the presence of abnormal PK resistant PrP (PrP^res^ -antibody Sha31 epitope YEDRYYRE). The number of PrP^res^ WB positive replicates corresponding to each round and each dilution are reported.

Infectivity titer (ID_50_ per mL of 10% brain homogenate) and seeding activity titer 50 (SA_50_ per mL of 10% brain homogenate) were estimated using the Spearman Karber’s limiting dilution titration method (most likely value). ND: not done. Incubation periods (in days) are shown as mean±standard deviation (SD) except when less than 100% of the animals developed clinical sign. In that case individual incubation period are presented.
